# The Impact of Immune Cells on the Skeletal Muscle Microenvironment During Cancer Cachexia

**DOI:** 10.3389/fphys.2020.01037

**Published:** 2020-08-31

**Authors:** Brandon N. VanderVeen, E. Angela Murphy, James A. Carson

**Affiliations:** ^1^Department of Pathology, Microbiology, and Immunology, School of Medicine, University of South Carolina, Columbia, SC, United States; ^2^AcePre, LLC, Columbia, SC, United States; ^3^Integrative Muscle Biology Laboratory, Division of Rehabilitation Sciences, College of Health Professions, University of Tennessee Health Science Center, Memphis, TN, United States

**Keywords:** extracellular matrix, macrophage, satellite cell, fibroblast, endothelium

## Abstract

Progressive weight loss combined with skeletal muscle atrophy, termed cachexia, is a common comorbidity associated with cancer that results in adverse consequences for the patient related to decreased chemotherapy responsiveness and increased mortality. Cachexia’s complexity has provided a barrier for developing successful therapies to prevent or treat the condition, since a large number of systemic disruptions that can regulate muscle mass are often present. Furthermore, considerable effort has focused on investigating how tumor derived factors and inflammatory mediators directly signal skeletal muscle to disrupt protein turnover regulation. Currently, there is developing appreciation for understanding how cancer alters skeletal muscle’s complex microenvironment and the tightly regulated interactions between multiple cell types. Skeletal muscle microenvironment interactions have established functions in muscle response to regeneration from injury, growth, aging, overload-induced hypertrophy, and exercise. This review explores the growing body of evidence for immune cell modulation of the skeletal muscle microenvironment during cancer-induced muscle wasting. Emphasis is placed on the regulatory network that integrates physiological responses between immune cells with other muscle cell types including satellite cells, fibroblast cells, and endothelial cells to regulate myofiber size and plasticity. The overall goal of this review is to provide an understanding of how different cell types that constitute the muscle microenvironment and their signaling mediators contribute to cancer and chemotherapy-induced muscle wasting.

## Introduction

Cachexia is a perilous comorbidity occurring with many chronic diseases that are defined by progressive weight loss, skeletal muscle atrophy, and the inability to be fully prevented or treated by nutritional support (Fearon et al., 2011). Adverse consequences resulting from cachexia include loss of functional independence, poor treatment outcomes, and a reduced quality of life (Bruera, 1997, Fearon et al., 2011). Cancer cachexia is a complex condition that is the most common cancer-associated comorbidity and occurs in ~50–80% of cancer patients (Baracos et al., 2018). To this end, the cachectic condition is linked to reduced responsiveness to anticancer therapy and increased mortality (Kazemi-Bajestani et al., 2016; Baracos et al., 2018; Sealy et al., 2020). There is inherent difficulty to examining the mechanistic drivers of cachexia, namely a large number of disruptions to systemic homeostatic regulation that occur with cancer, the inherent heterogeneity of different cancers, and the patient’s underlying health condition. Moreover, skeletal muscle mass is sensitive to the several systemic disruptions associated with cancer including insulin resistance, chronic inflammation, anorexia, and hypogonadism (Baracos et al., 2018). There also has been significant interest in investigating tumor derived factors that can directly drive skeletal muscle catabolism, and numerous factors have been identified (Biswas and Acharyya, 2020). Despite our vastly improved mechanistic understanding of cancer-induced muscle wasting through established and well-defined preclinical cachexia and *in vitro* atrophy models, the complexity and heterogeneity of cancer cachexia have hindered the development of effective treatments for the cancer patient (Anderson et al., 2017). Additionally, mechanistic studies have not historically considered the potential additive effects of cancer and chemotherapy on the mechanisms inducing cachexia, and we are only beginning to understand the implications of this interaction for the management of cachexia (Barreto et al., 2016a,b; Bozzetti, 2020).

Systemic and local inflammation accompany many different conditions that produce skeletal muscle metabolic plasticity, growth, and atrophy, and a regulatory role for inflammation in these processes has been widely investigated for decades (Tidball, 1995; Deans and Wigmore, 2005). Additionally, transient increases in systemic inflammation and intrinsic skeletal muscle inflammatory signaling can occur with exercise and has been linked to many important muscle adaptations (Febbraio et al., 2004; Deyhle et al., 2015). Chronic systemic inflammation is a widely investigated driver of muscle wasting through its direct effects on skeletal muscle (Baracos et al., 2018), and its ability to induce other systemic disruptions that can ultimately regulate skeletal muscle mass, such as insulin resistance and hypogonadism (Wu and Ballantyne, 2017). The ability to regenerate from injury is a recognized property of healthy skeletal muscle, and immune cells have a well-established role in this regenerative process (Howard et al., 2020). While inflammation’s contribution to initiating and accelerating cancer cachexia has been widely investigated (Evans et al., 2008; Carson and Baltgalvis, 2010), a major focus of this research has centered on circulating inflammatory mediators and how they directly regulate muscle intracellular signaling to disrupt protein turnover and metabolism to drive wasting (Talbert et al., 2018). To this end, significant gaps remain in our understanding of other aspects of the complex relationship between the immune system and the regulation of skeletal muscle mass. Additional research is warranted to delineate the capacity for inflammation to regulate signaling between different cell types in skeletal muscle that is involved in maintaining metabolic and protein turnover homeostasis. Immune cells comprise 2–6% of skeletal muscle’s cell population, but maintain a well-established role in skeletal muscle homeostasis, especially macrophages (MΦ; Tidball, 2002; Reidy et al., 2019a). While the understanding of the MΦ’s role in skeletal muscle repair and remodeling is well-appreciated, there is strong evidence for both T-cells and neutrophils in the maintenance of skeletal muscle MΦ function and overall skeletal muscle plasticity (Frenette et al., 2002; Tidball, 2005; Dumont et al., 2008; Schiaffino et al., 2017; Tidball, 2017; Deyhle and Hyldahl, 2018). Despite the importance of immune cell activity in muscle plasticity and aging (Reidy et al., 2019a), our understanding of immune cell involvement in cancer‐ and chemotherapy-induced muscle wasting is just emerging.

The potential for cancer to disrupt tightly regulated interactions between cell types in the skeletal muscle microenvironment continues to develop and be appreciated (Talbert and Guttridge, 2016). Skeletal muscle microenvironment interactions have established functions in muscle response to regeneration from injury, growth, aging, overload-induced hypertrophy, and exercise (Morgan and Partridge, 2020). Furthermore, there has been extensive investigation into the importance and regulation of satellite cell proliferation and differentiation, angiogenesis, and extracellular matrix (ECM) remodeling after muscle injury and with aging (Tidball and Wehling-Henricks, 2007; Xiao et al., 2016; Ceafalan et al., 2018; Yang and Hu, 2018). These adaptive processes are often coupled to local inflammatory responses initiated by remodeling stimuli. These inflammatory responses are subjected to precise temporal regulation and if this response is altered, muscle remodeling can be either attenuated or blocked (Howard et al., 2020). Moreover, systemic and intrinsic stimuli can induce MΦs to initiate signaling that regulates muscle fibroblasts, satellite cells, endothelial/vascular cells, as well as within the myofiber (Tidball, 2002; Arnold et al., 2007; Fry et al., 2014). Inflammatory signaling can impact several cell types located in the muscle microenvironment leading to altered myofiber protein synthesis (Gao et al., 2017) and mitochondrial quality control (Gomez-Cabrera et al., 2016; Fix et al., 2018), which are known drivers of muscle wasting with cancer. Additionally, muscle fibrosis and dysregulation of the ECM, of which immune cells play a central regulatory role, have been reported in cachectic skeletal muscle from pancreatic cancer patients (Judge et al., 2018; Nosacka et al., 2020). Therefore, this review explores the growing body of evidence for immune cell modulation of the skeletal muscle microenvironment during cancer-induced muscle wasting. Emphasis is placed on the regulatory network that integrates physiological responses between immune cells with other muscle cell types including satellite cells, fibroblast cells, and endothelial cells to regulate myofiber size and plasticity. The overall goal of this review is to provide an understanding of the potential for different cell types that constitute the muscle microenvironment that can contribute to cancer-induced muscle wasting. Specific attention is given to MΦ, neutrophil, and T-cell’s regulation of the communication between several cell types in the muscle microenvironment that could promote cancer-induced myofiber catabolism and metabolic dysfunction. The current understanding of chemotherapy as an underlying pathology that could disrupt immune cell interaction with the skeletal muscle microenvironment also is discussed.

## Cancer and Chemotherapy-Induced Cachexia Overview

### Mechanisms of Cancer-Induced Cachexia

Skeletal muscle loss is a critical manifestation of cancer cachexia (Argiles et al., 2010). While our mechanistic understanding of cancer-induced muscle wasting continues to develop and potential therapeutic targets to treat the condition have been identified, both the complexity of the underlying disease and the multiple mechanisms responsible for the maintenance of skeletal muscle homeostasis have clouded our ability to understand the primary drivers of this catabolic condition. While there may still be hope for a “master regulator” of cancer-induced muscle atrophy (Lecker et al., 2004), the heterogeneity of cancer, pre-existing conditions of the patient, and equivocal findings between preclinical cachexia models have led to a host of potential drivers of the cachectic condition that are too broad to describe in detail here. However, this topic has been extensively reviewed (Argiles et al., 2014, 2015; Carson et al., 2016; Vanderveen et al., 2017; Baracos, 2018). Skeletal muscle responds to a multitude of systemic cues that are vital for whole body homeostasis, and the muscle fiber responds to global stimuli including disuse, increased activity, aging, and metabolic mediators (Atherton et al., 2016), which can all be altered with cancer and chemotherapy. Furthermore, due to these varied physiological and functional demands, muscle fibers differ in contractile and metabolic properties, and these properties can impact how the muscle fiber responds to the cancer environment (Carson et al., 2016; Vanderveen et al., 2017). The complexity of the muscle’s physiological response to cancer and chemotherapy is also impacted by the myofiber’s interaction with the ECM, vasculature, and response to local inflammation, which is examined in this review.

Despite the identified complexities in understanding muscle mass regulation with cancer, muscle mass maintenance requires a balanced regulation of protein turnover, which is maintained by anabolic and catabolic signaling pathways. Cancer induces fundamental disruptions to the homeostatic regulation of the protein turnover that has negative consequences for skeletal muscle function and cellular metabolism (Baracos et al., 2018; Counts et al., 2020). Cachexia drives overall muscle mass loss even with adequate nutrient intake (Fearon et al., 2012a; Porporato, 2016), and results in suppressed protein synthesis and/or increased degradation. Muscle protein synthesis and breakdown can be regulated by various stimuli including nutrient availability, hormones, mechanical loading, and metabolic stress, which can all be disrupted by cancer and potentially chemotherapy (Glass, 2005; Powers et al., 2005; Zhang et al., 2007; Egerman and Glass, 2014). Active areas of investigation for the disruption of protein turnover with cancer have focused on excessive protein breakdown by the ubiquitin proteasome system (UPS) and disrupted autophagy regulation (McClung et al., 2010; White et al., 2011; Fearon et al., 2012b; Penna et al., 2013; Luo et al., 2014; Aversa et al., 2016). Critical intracellular regulator networks in skeletal muscle exert tight control of these processes but can be disrupted by cancer and chemotherapy. This skeletal muscle regulatory nexus involves Akt, mTORC1, FOXO3A, and AMPK signaling, which responds to growth factors, mechanical stimuli (i.e., stretch and contraction), cellular energy levels (AMP: ATP), nutrients (amino acids), and inflammation (Carson, 1997; Carson and Wei, 2000; Glass, 2005; Judge et al., 2014). These mechanisms exert control over cell growth through regulation of protein synthesis (e.g., transcription and translation), protein degradation (e.g., UPS) autophagy, and metabolism (Laplante and Sabatini, 2009). Disruption to this signaling network in cachectic skeletal muscle also has been linked to cancer-induced mitochondrial dysfunction (Carson et al., 2016; Vanderveen et al., 2017, 2019; Fix et al., 2019). Muscle mitochondrial dysfunction continues to be extensively investigated as an underlying mechanism for the initiation and progression of cancer-induced muscle wasting. However, understanding how cancer disrupts the normal dynamic regulation of muscle protein turnover remains an active area of inquiry.

Cancer-induced systemic inflammation and the increased production of specific cytokines have been extensively investigated as an underlying driver of cancer cachexia (Argilés et al., 2019). Both adipose tissue and skeletal muscle catabolism can be induced by exacerbated inflammation. Examination of cachectic cancer patients and preclinical models of cachexia has identified interleukin 6 (IL-6), tumor necrosis factor α (TNF-α), TNF-like inducer of apoptosis (TWEAK), TNF receptor (TNFR)-associated factor 6 (TRAF6), interferon gamma (INF-γ), and leukemia inhibitory factor (LIF) as mediators of cancer-induced muscle wasting (White et al., 2013; Hetzler et al., 2015; Zimmers et al., 2016; Yakovenko et al., 2018; Argilés et al., 2019). These cytokines can induce several intracellular pathways including the nuclear factor-κB (NF-κB) pathway, p38 mitogen-activated protein kinase (MAPK) pathway, and the Janus kinase/signal transducer and activator of transcription (JAK/STAT) pathway. Notwithstanding the identification of several different inflammatory mediators in cachexia, we lack understanding of how these signaling pathways interact or overlap with other physiological signaling within the muscle microenvironment to regulate muscle catabolism with cancer. Due to this complexity barriers remain for implementing therapeutic strategies that target an individual signaling cascade to preserve skeletal muscle mass in the cancer patient. Moreover, there are currently no FDA-approved treatments for cancer cachexia. That said, there remains strong therapeutic potential for nutritional, anti-inflammatory, and lifestyle management strategies to mitigate cachexia severity and improve patient life quality.

### Mechanisms of Chemotherapy-Induced Cachexia

Cachexia’s prevalence with cancer is often dependent on cancer type and stage of the disease; however, the functional deficits with chemotherapy are consistent and pervasive (Barreto et al., 2016a; Bozzetti, 2020). Along with nausea, emesis, and anorexia, weakness and fatigue remain among the most commonly reported off-target effects of chemotherapy (Dahele et al., 2007). Our understanding of chemotherapy’s deleterious off-target effects on skeletal muscle has improved over the last decade highlighting a key role for metabolic and DNA/cell stress (Gilliam et al., 2009; Barreto et al., 2016b; Morton et al., 2019; Sougiannis et al., 2019). Targeting the generation of mitochondrial reactive oxygen species (ROS), by exercise or antioxidants, has shown promise in mitigating Doxorubicin (DOX)-induced skeletal muscle dysfunction (Smuder, 2019; Montalvo et al., 2020), and targeting the activation of MAPKs with ACVR2B/Fc and MEK1 inhibitors has been proposed to alleviate 5-FU induced mitochondrial dysfunction (Barreto et al., 2016b, 2017). Inflammatory signaling has been demonstrated to regulate chemotherapy-induced E3 ligase activation through modulating TNF-α (Gilliam et al., 2009). Interestingly, while conclusive mechanisms involving intrinsic skeletal muscle inflammatory signaling with chemotherapy are lacking, leukopenia with chemotherapy has been well-established (Kvinnsland, 1999; Yamanaka et al., 2007; Shitara et al., 2009; Baechler et al., 2010; Han et al., 2012; Abraham et al., 2015). This naturally invites intrigue into understanding how chemotherapy-induced immunosuppression can converge with cancer-associated chronic inflammation to disrupt the skeletal muscle microenvironment, which can lead to wasting, reduced life quality, poor treatment outcomes, and subsequent reduced survival. While again there are currently no approved treatments for chemotherapy’s off-target effects on muscle, there remains similar strong therapeutic potential for nutritional, anti-inflammatory, antioxidant, and/or lifestyle management to mitigate chemotherapy-induced toxicities.

The identification of immune checkpoints, specifically PD-1/PDL1 and CTLA-4, has led to the use of specific inhibitors, which have greatly improved cancer therapies (Haanen and Robert, 2015). These drugs improve the anti-tumor capacity of T-cells and potentially natural killer (NK) cells by blocking the ligand/receptor binding induced by cancer cells that cause T-cell inactivation (Curran et al., 2010; Pesce et al., 2019). Similar to other cancer treatments, patients receiving immune check point inhibitors (ICPIs) that suffer from cachexia have reduced survival when compared to patients who maintain their weight (Chu et al., 2020; Roch et al., 2020). Whether ICPIs can induce cachexia has not been established; however, body weight loss in aged mice with mesothelioma tumors was exacerbated by immunotherapy, but whole body macrophage depletion reduced tumor mass and body weight loss (Duong et al., 2018). As using ICPIs become increasingly more common as a cancer treatment, understanding the impact of cachexia and the role of ICPI in cachexia progression remains an intriguing area for future inquiry.

### Overlap and Differences of Cancer and Chemotherapy-Induced Cachexia

Developing an understanding of the distinct contributions of cancer and chemotherapy to muscle wasting and dysfunction has been at the forefront of cachexia related mechanistic inquiry; however, understanding the potential synergism or negation of specific mechanisms with both cancer and chemotherapy would improve our understanding of cancer patient’s condition. To date, few studies have directly investigated the effect of cancer and chemotherapy on skeletal muscle which leads to cachexia. Barreto et al. (2016a) identified the common pathways activated by C26-induced cachexia and chemotherapeutic Folfiri [5-fluorouracil (5FU), leucovorin, irinotecan]-induced cachexia. This study highlighted a common downregulation of metabolic and structure proteins with particularly striking changes to mitochondrial function (Barreto et al., 2016a). Interestingly, only Folfiri induced neurological damage, while only C26 was associated with exercise intolerance. Others have demonstrated that TNF-α plays a key role in cancer and DOX-induced skeletal muscle dysfunction (Hardin et al., 2008; Gilliam et al., 2009, 2011). While disrupted protein turnover has been a central regulator of cancer-induced cachexia (White et al., 2013; Montalvo et al., 2018; Counts et al., 2020), there is little evidence to suggest chemotherapy impacts muscle in this same way.

## Immune Cells

### Monocytes

#### Monocyte’s Role in Physiology

**Figure 1:**
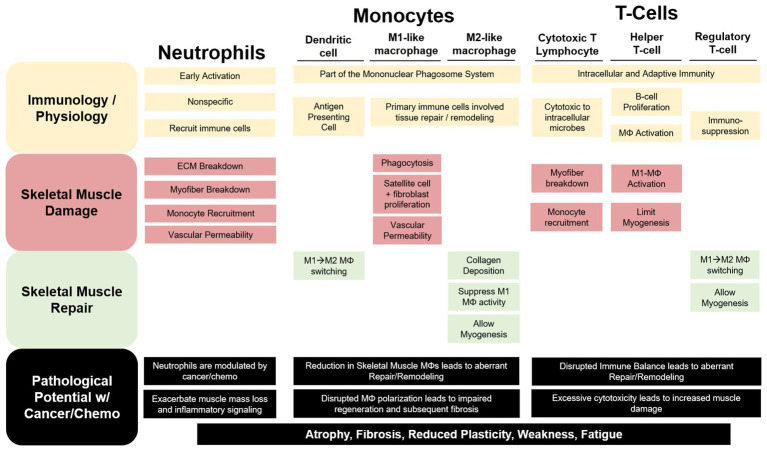
The contribution of immune cells to skeletal muscle repair and remodeling. Neutrophils, monocytes, and T-cells have both unique and redundant processes that aid in resolving a skeletal muscle insult. Neutrophils are recruited early and exact a robust, non-specific response that can exacerbate damage to the myofiber and extracellular matrix (ECM). Cytotoxic T-lymphocytes (CTLs) have been demonstrated to perform similar functions to neutrophils in the early stage of the damage response. Additionally, Neutrophils and CTLs recruit inflammatory (M1-like) macrophages (MΦ) to help phagocytize damaged tissue for removal while releasing pro-inflammatory mediators to stimulate satellite and fibroblast cell proliferation and increase vascular permeability. As repair progresses anti-inflammatory, profibrotic (M2-like), MΦs predominate skeletal muscle allowing for myogenesis and collagen deposition. Regulatory T-cells and dendritic cells (DC) aid in the MΦ phenotype switching contributing to the switch from a pro-inflammatory to an anti-inflammatory skeletal muscle microenvironment. Cancer and chemotherapy have been demonstrated to modulate each of these immune cells uniquely in circulation or other non-muscle tissue; however, we are limited in our understanding of their roles in cancer-associated wasting. We can speculate that these disruptions to these cells within the skeletal muscle microenvironment would lead to muscle mass loss and increased fibrosis contributing to reduced muscle plasticity, weakness, and fatigue.

The mononuclear phagocyte system (MPS) includes circulating monocytes, monocyte derived MΦs, and dendritic cells (DC; Gordon and Taylor, 2005; Hume, 2006). As their name implies, they share a mononuclear structure and play key roles in tissue defense and homeostasis through phagocytosis of old or damaged materials as well as invading pathogens (Murphy and Weaver, 2016). Upon insult, naïve monocytes proliferate in the bone marrow in response to chemokines, most notably monocyte chemoattractant 1 (MCP-1), to infiltrate the damaged/infected tissue to phagocytize pathogens or damaged material (Hume, 2006; Capoccia et al., 2008). Once outside vasculature, monocytes most commonly differentiate to either DC or MΦ; however, tissues also house resident, nascent, and self-renewing DCs and MΦs (Van Furth and Cohn, 1968; Hoeffel and Ginhoux, 2018). It is also important to note that MΦs have been demonstrated to play both an anti‐ and pro-inflammatory roles in both innate and adaptive immunity depending on the stimuli; however, MΦs are the primary phagocytic effector cell of the MPS (Gordon and Taylor, 2005). While DCs share a morphology with undifferentiated monocytes and MΦs, they primarily function as antigen presenting cells (APC) which activate naïve T-cells within the adaptive immune response (Mellman, 2013; Murphy and Weaver, 2016; Abbas et al., 2018; Figure 1).

Unfortunately, due to overlapping functions and shared cell surface markers, characterizing and assessing the different subsets of these monocytes are contentious and equivocal (Gordon and Taylor, 2005; Guilliams et al., 2014). The most conserved monocyte cell surface marker between humans and rodents are CX3C chemokine receptor (CX_3_CR1) and cluster of differentiation (CD) 11b. These markers vary in expression based on the phenotype of the monocyte. For example, resident or “patrolling” monocytes will have a higher expression of CX_3_CR1 compared to circulating or inflammatory monocytes, and the expression of Ly6C in mice is a spectrum from resident (low-none) to inflammatory or circulating (med-high; Gordon and Taylor, 2005). However, undifferentiated Ly6C^High^ monocytes have been described in healthy uninjured tissue while Ly6C^Low^ survey the endothelium (Guilliams et al., 2014). DCs share the CX_3_CR1 and CD11b receptors with other monocytes; however they differ in their expression of CD11c and major histocompatibility complex (MHC) II and can even express common T-lymphocyte markers CD4 and CD8 (Sato and Fujita, 2007). The varying degrees of these cell surface markers help define physiology and function as well as their myeloid or lymphoid origin/maturation (Sato and Fujita, 2007; Mellman, 2013). All murine MΦs are said to express CD68 and F4/80 irrespective of phenotype. Mature/differentiated MΦs are not common in the circulation but are found as naïve monocytes until they extravasate. Resident self-renewing MΦs are commonly considered M0 and lack, or have low expression of CD11c, MHCII, CD80, or CD206. These resident M0-like MΦs can either remain nascent or be polarized to increase expression of CD11c and/or MHCII to become a M1-like pro-inflammatory, phagocytic MΦ (Shapouri-Moghaddam et al., 2018; Orecchioni et al., 2019). While these resident cells are able to polarize to an M1-like phenotype, this classification is more common among infiltrating monocytes (Ly6C^High^). Furthermore, these M1-like MΦs are plastic and their expression of pro-inflammatory cell surface markers and release of pro-inflammatory cytokines can be suppressed with a corresponding increase in the expression of CD206 and/or CD163 and release of anti-inflammatory cytokines resulting in an M2-like anti-inflammatory, pro-fibrotic MΦ (Gordon and Taylor, 2005; Mosser and Edwards, 2008; Wynn et al., 2013). MΦs are plastic and responsive to extrinsic stimuli; however, autocrine/paracrine MΦ signaling can regulate their phenotype through feedback or feedforward mechanisms. Furthermore, the M1/M2 dichotomy does not adequately described the nature and function of the MΦ and should be considered a spectrum rather than an “either/or” (Mosser and Edwards, 2008). This classification of cell surface markers and function is not exhaustive and is often contentious, which has been previously reviewed (Hume, 2006; Mellman, 2013; Guilliams et al., 2014).

#### Monocyte’s Role in Skeletal Muscle Repair and Remodeling

MΦs have received significant attention in skeletal muscle physiology given their central role in repair and remodeling, and thus our understanding of skeletal muscle MΦs primarily comes from studies investigating repair, regeneration, and remodeling following unloading/reloading-, eccentric contraction-, or cardiotoxin-induced damage (St Pierre and Tidball, 1994; Tidball, 2005, 2017; Cote et al., 2013). Early in skeletal muscle repair, there is an influx of circulating monocytes that differentiate to a phagocytic and pro-inflammatory M1-like MΦ to aide in the removal of damaged tissue. Additionally, they recruit additional inflammatory monocytes by MCP-1, promote vascularization by HIF-1α, stimulate satellite cells proliferation, and inhibit satellite cells differentiation by IL-6 and TNF-α (Frenette et al., 2003; Arnold et al., 2007; Capoccia et al., 2008; Novak et al., 2014; Liu et al., 2017; Ceafalan et al., 2018). The plasticity of MΦs, however, allows for phenotypic flexibility and within 7 days following damage a more M2-like profibrotic anti-inflammatory phenotype predominates (St Pierre and Tidball, 1994; Tidball and Wehling-Henricks, 2007; Hammers et al., 2015). These M2-like MΦs then promote angiogenesis by secreting vascular endothelial growth factor (VEGF) and ECM remodeling by secreting TGFβ and matrix metalloproteases (MMP) 2 and 9 while allowing for myogenesis through suppression of TNF-α release (De Santa et al., 2018).

Disrupted skeletal muscle repair with aging and chronic disease has been described, though mechanistic explanations often focus on sex hormones, satellite cells, or intrinsic structural dysmorphias. Despite the understood immune changes with aging and disease and the established roles of monocytes in skeletal muscle homeostasis, our understanding of the relationship between the immune system and musculoskeletal system under such conditions is in its infancy (Cui et al., 2019; Reidy et al., 2019a). Immunological investigation into muscular dystrophy demonstrated that dystrophic mice had profound increases in total muscle MΦs and targeting this infiltration can mitigate disease progression (Mojumdar et al., 2014; Villalta et al., 2015). Additionally, reloading aged skeletal muscle had a blunted hypertrophy response associated with a lower number of M1-like MΦs at baseline and blunted M1-like MΦ infiltration (early) and M2-like MΦ transition (late; Reidy et al., 2019b). To this end, it is important to note that the mean age of cancer patients is ~65 years and it may be that these changes with aging overlap with cancer and chemotherapy-induced changes to skeletal muscle MΦs (Dunne et al., 2019).

#### Monocytes Role in Cancer and Chemotherapy-Induced Cachexia

To date, few papers have directly investigated the changes to skeletal muscle monocytes or MΦs with cancer and/or cancer-associated wasting (Inaba et al., 2018; Costamagna et al., 2020). Interleukin-4 (IL-4), a potent stimulator of M2-like MΦs, was shown to attenuate skeletal muscle wasting in the C26 mouse model of cachexia associated with reduced satellite cell accumulation and increased skeletal muscle CD206 protein expression (Costamagna et al., 2020). Protein analysis showed no significant differences between control and C26-tumor bearing mice with F4/80, and CD206 (marker of M2-like MΦs) appears lower in C26 mice but did not achieve statistical significance. While elevated circulating IL-6 and LIF is diagnostic in the C26 model, Inaba et al. (2018) demonstrated reduced skeletal muscle MΦ and neutrophil number without the loss of satellite cell proliferative or differentiative abilities that others have demonstrated (Penna et al., 2010). Evidence of disrupted skeletal muscle regeneration – a process reliant on skeletal muscle MΦs – with cachexia has been demonstrated (Devine et al., 2015; Judge et al., 2018). Whether cachexia leads to muscle degeneration or fibrosis without a damaging insult is equivocal likely due to the heterogeneity of tumor type and preclinical model; however, reduced specific force (force per unit area) with cachexia has been demonstrated across several preclinical models, corresponding with the severity of weight loss (Jaweed et al., 1983; Gorselink et al., 2006; Murphy et al., 2012; Roberts et al., 2013; Ramage and Skipworth, 2018; Vanderveen et al., 2018). The role of monocytes/MΦs in these aberrations has been merely speculative highlighting a need for additional studies examining changes to MΦs in skeletal muscle with cachexia progression. There is, however, intriguing evidence suggesting the immunosuppressive nature of common chemotherapeutic DOX may be disrupting skeletal muscle MΦ activation and polarization leading to blunted skeletal muscle repair and remodeling (Huang et al., 2017). Interestingly, disrupted skeletal muscle inflammatory signaling with DOX and/or 5FU has not been evident, despite the cytotoxic nature of the drugs. Current evidence remains focused on DNA damage or stress sensors, p38 and Erk1/2, mitochondrial damage, oxidative stress, and autophagy with these chemotherapeutics (Barreto et al., 2016a; Ballaro et al., 2019; Hiensch et al., 2019; Bozzetti, 2020).

### Neutrophils

#### Neutrophils Role in Physiology

Neutrophils belong to a class of leukocytes characterized by their polymorphic nuclear shape and their early recruitment during acute inflammation (Kolaczkowska and Kubes, 2013; Stackowicz et al., 2019). Neutrophils make up roughly ~50% of circulating leukocytes in healthy individuals (~15% in mice) and are the first line of defense against bacterial infection (Kolaczkowska and Kubes, 2013). Interestingly, neutrophils share a precursor cell with monocytes and originate in the bone marrow but rely on granulocyte colony-stimulating factor (G-CSF) for simulation as opposed to monocyte colony-stimulating factor (M-CSF). Classifying neutrophils is much more straight forward compared to monocytes; however, there remains variety among immunologists. Neutrophils commonly express CD11b and CD16 (Fujimoto et al., 2000); however, recently the use of Ly6G and Gr-1 has been used to differentiate neutrophils from other leukocytes (Lee et al., 2013; Romee et al., 2013). Neutrophils kill/eliminate pathogens through phagocytosis, degranulation, ROS production, and/or neutrophil extracellular traps, which have been well-reviewed (Kolaczkowska and Kubes, 2013; Mayadas et al., 2014). The granules within neutrophils contain lysozymes, collagenases, and elastases, which serve the function of damaging/killing foreign material. Unfortunately, neutrophils are early and robust in their response and their lack of specificity often damages the host tissue along with the pathogen (Faurschou and Borregaard, 2003; Mayadas et al., 2014). While they play an important role in acute cytotoxicity, neutrophils release a myriad of cytokines and oxidative factors to initiate and potentiate the immune response depending on the stimuli. Neutrophils are often replaced by MΦs following the initial stages of damage or infection primarily through neutrophil release of pro-inflammatory cytokines and myeloperoxidases (MPOs).

#### Neutrophils Role in Skeletal Muscle Repair and Remodeling

The majority of studies investigating the musculoskeletal and immune system relationship have focused on monocytes/MΦs; however, there is evidence that neutrophils play a key role in the clearance of damaged/dead tissue and recruitment of MΦs (Tidball et al., 1999; Pizza et al., 2005). Historically, ischemia/reperfusion-induced skeletal muscle damage resulted in a dramatic increase influx of neutrophils while MΦs play a more minor role (Korthuis et al., 1988; Smith et al., 1989; Kanwar et al., 1998); however, the role for neutrophils in eccentric contraction-induced damaged also has been described (Frenette et al., 2002; Pizza et al., 2005). While the plasticity of MΦ polarization is a key regulator of the switch from the initial skeletal muscle damage response to repair/regeneration, neutrophils appear to play a significant role only in the initial damage response. To this end, persistent neutrophil infiltration has been demonstrated to delay skeletal muscle repair and absence of neutrophil accumulation mitigates signs of skeletal muscle damage (Pizza et al., 2005). Further, elevated neutrophils have been demonstrated to exacerbate joint damage with arthritis, and some evidence suggests that neutrophils can induce or exacerbate skeletal muscle damage (Dumont et al., 2008). The induction of neutrophils with exercise and ischemia/reperfusion-induced damage is transient, and the decrease in the relative abundance of neutrophils is accompanied by a polarization switch of MΦs to an M2-like profibrotic/anti-inflammatory phenotype for remodeling (Tidball et al., 1999; Frenette et al., 2003; Tidball, 2005; Tidball and Villalta, 2010). Interestingly, the acute changes in the genetic profile of skeletal muscle with exercise reflect similar changes in blood neutrophils and circulating inflammatory cytokines (Broadbent et al., 2017). While the role of neutrophils in skeletal muscle physiology requires significant work, there is evidence to support that neutrophils exacerbate the damage response to perpetuate the acute inflammatory stimuli necessary for repair and remodeling.

#### Neutrophils Role in Cancer and Chemotherapy-Induced Cachexia

Neutropenia has been shown to be a strong prognostic indicator for survival in cancer patients undergoing chemotherapy (Kvinnsland, 1999; Yamanaka et al., 2007; Han et al., 2012; Abraham et al., 2015). Regarding cancer, the circulating neutrophil-to-lymphocyte ratio (NLR) is also a strong prognosticator for all-around survival (Grecian et al., 2018). In general, tumors produce G-CSF and can increase circulating neutrophils (Jablonska et al., 2017) while chemotherapy reduces circulating neutrophils (Shitara et al., 2011). These findings appear dependent on cancer type and chemotherapy as some investigators have reported decreases in neutrophils with cancer and increases with chemotherapy. To our knowledge, the impact of neutrophil changes with cancer and chemotherapy on skeletal muscle has not been investigated. We can speculate, however, that an increase in circulating neutrophils with cancer may initiate and/or potentiate skeletal muscle degeneration or mass loss while neutropenia may blunt the homeostatic balance of skeletal muscle repair and remodeling. It is understood that a proper balance of pro‐ and anti-inflammatory effectors is essential for skeletal muscle homeostasis and the undulating changes to neutrophils with cancer and cancer therapies warrants investigative inquiry.

### Lymphocytes: T-Cells

#### T-Cells Role in Physiology

Lymphocytes are key regulators of the immune response and play a central role in adaptive immunity. Lymphocytes are characterized as B (B-cells) or T lymphocytes (T-cells) based on their function and origin (B-Bone Marrow; T-Thymus) rather than their appearance as they both maintain a similar morphology. Similar to other immune cells, B-cells release inflammatory mediators to regulate an immune response, but their primary function is often focused on extracellular pathogens and antibody production (Murphy and Weaver, 2016). Their role as an immune modulator with skeletal muscle has not been well-characterized and requires additional work; therefore, this review will focus on the known roles of T-cells. T-cells are key regulators of cellular immunity either through eliminating intracellular microbes or the activation of other immune cells including MΦs (Abbas et al., 2018). The classification of T-cells is extensive and depends again on their cellular function and role in the immune response. Classic characterization aligns with CD3+CD4+ helper T-cells (Th), CD3+CD4+CD25+FoxP3+ T regulatory cells (Tregs), and CD3+CD4-CD8+ cytotoxic T lymphocytes (CTL). Historically, Th cells regulate B-cell differentiation in immune tissue and activate resident or local MΦs for cellular immunity and can be further classified into Th1, Th2, and Th17 (Th3, Th9, Th22, and TFH cells have also been described; Hirahara and Nakayama, 2016). Th1 cells regulate intracellular phagocytosis by activating MΦs through the release of pro-inflammatory IFNγ, while Th2 cells can release IL-4 and IL-13 to modulate the MΦ phenotype switching to an M2-like anti-inflammatory phenotype (Shapouri-Moghaddam et al., 2018; Mazzoni et al., 2019). Th17 cells are only beginning to be understood; however, they produce known pro-inflammatory cytokines IL-17 and IL-6, and have an emerging role in auto-immunity (Bi et al., 2007; Mazzoni et al., 2019). CD8+ CTL are aptly named as they provide intracellular immunity against pathogens by inducing cell death in cells that are not accessible or recognized by antibodies (Mittrucker et al., 2014). While Th cell activation and proliferation play a key role in antibody-mediated immune function in circulation, CTLs are the primary effectors of the intracellular immune response (Abbas et al., 2018). Tregs, as their name suggests, play a key role in regulating the immune response primarily through immune suppression and regulate the M1-like to M2-like MΦ phenotype switch during remodeling (Hori et al., 2003). The different subsets of lymphocytes are vast, and each play an important role in the immune system; however, for our purposes we will highlight the literature examining the roles of Th, Tregs, and CTLs in skeletal muscle physiology, repair, and remodeling and their potential role in modulating muscle wasting with cancer and chemotherapy.

#### T-Cells Role in Skeletal Muscle Repair and Remodeling

The role of T-cells in skeletal muscle repair and remodeling has been recently reviewed and described briefly below (Schiaffino et al., 2017; Deyhle and Hyldahl, 2018). Tregs primarily function in immune suppression, which is necessary during the later stages of skeletal muscle repair/remodeling, while CTLs and Th cells may potentiate the early damage response (Zhang et al., 2014; Deyhle and Hyldahl, 2018). Tregs release anti-inflammatory cytokine IL-10 to regulate M1-to-M2 like MΦ phenotype switching during muscle repair following injury (Deng et al., 2012; Schiaffino et al., 2017; D’Alessio et al., 2019). Tregs also have been shown to stimulate proliferation of satellite cells through the release of Amphireglin (AREG) – a key regulator of immunity and tissue repair (Burzyn et al., 2013; Arpaia et al., 2015; Castiglioni et al., 2015; Zaiss et al., 2015). The mechanisms of Treg’s regulation of skeletal muscle repair are still being unearthed; however, the induction of IL-33 with injured skeletal muscle appears the likely candidate for the increased abundance of Tregs and Treg-associated AREG (Kuswanto et al., 2016). Our understanding of CD8+ CTLs role in skeletal muscle repair centers around potentiating the immune response through its own infiltration into damaged muscle and releasing pro-inflammatory cytokines, namely MCP-1 (Zhang et al., 2014). Rag1 deficient mice that lack both T‐ and B-cells, but maintain intact MΦs, had delayed skeletal muscle repair following cardiotoxin; however, reintroduction of both CD4+ and CD8+ T-cells rescued the repair process timeline (Fu et al., 2015). These investigations primarily focused on the impact of T-cells on the activation and proliferation of satellite cells, which is necessary for the repair process.

#### T-Cells Role in Cancer and Chemotherapy-Induced Cachexia

CTLs and Th cells appear to play a role in the pathological damage response in Duchenne’s muscular dystrophy (DMD), while there is promise for manipulating Tregs to mitigate excessive skeletal muscle damage (Spencer et al., 2001; Villalta et al., 2015). Similar to neutrophils, there has not been investigative inquiry into the role of T-cells in cancer or chemotherapy-induced skeletal muscle wasting; however, T-cells have been investigated extensively for their role in immune suppression during cancer progression and are promising therapeutic targets given their role in cytotoxicity/cellular immunity (Naito et al., 1998; Almand et al., 2001). To this end, tumor infiltrating lymphocytes (TILs) and *ex vivo* expansion of T-cells provides important prognosis information (Walsh et al., 2005; Morgan et al., 2006). Interestingly, CD8+ T-cells have been demonstrated to induce skeletal muscle wasting during infection-associated cachexia as shown by improved body weight maintenance in CD8+ T-cell null mice (Baazim et al., 2019). However, mechanisms underlying infection-associated cachexia may be distinct from cancer-associated cachexia. The most promising study was conducted by Wang et al. (2008) showing infusion of CD4+CD44^Low^ naïve T-cells could attenuate muscle mass loss associated with ameliorating cancer-associated lymphopenia. Additionally, there is evidence of an inverse relationship between body weight and NLR – as NLR increases, body weight decreases – highlighting that this ratio may be an intriguing surrogate for the immune milieu responsible for cachexia progression (Derman et al., 2017). Given the important role of T-cells in cancer progression, treatment, and proposed role in muscle mass maintenance, studies aimed at understanding each T-cell’s role in cancer and chemotherapy-associated cachexia are needed.

### Other Immune Cell Modulators

While the primary focus of this review is to highlight the impact of monocytes, neutrophils, and T-cells on the skeletal muscle microenvironment, other immune cells involved in cancer’s immune disruptions should not be neglected as their role in skeletal muscle homeostasis is merely less understood. These cells include, but are not limited to, B-cells, NK cells, myeloid derived suppressor cells (MDSC), and mast cells (Murphy and Weaver, 2016). The role of these various cell types in the pathogenesis of DMD has been reviewed (Madaro and Bouche, 2014). B-cells were recently shown to modulate muscle weakness during dermatomyositis through the regulation of IFNγ (Radke et al., 2018). Mast cells aggregate and proliferated in sites of muscle necrosis following damage and can restore vascular permeability during myositis (Gorospe et al., 1996; Yokota et al., 2014); however, little is known about their role with muscle wasting with cancer and chemotherapy. Interestingly, MDSCs were shown to be increased with cancer thought to contribute to global disruptions in energy metabolism contributing to cachexia progression (Cuenca et al., 2014). A correlative increase of IL-17 and MDSCs were both associated with nutritional impairments with gastrointestinal cancer which was proposed to contribute to cachexia (Yazawa et al., 2013). Furthermore, the metabolic cost of myelopoiesis, mainly increased MDSCs, may also contribute to the metabolic abnormalities seen with cancer and chemotherapy (Sica and Strauss, 2017).

## Skeletal Muscle Microenvironment

### Myofiber Overview

**Figure 2:**
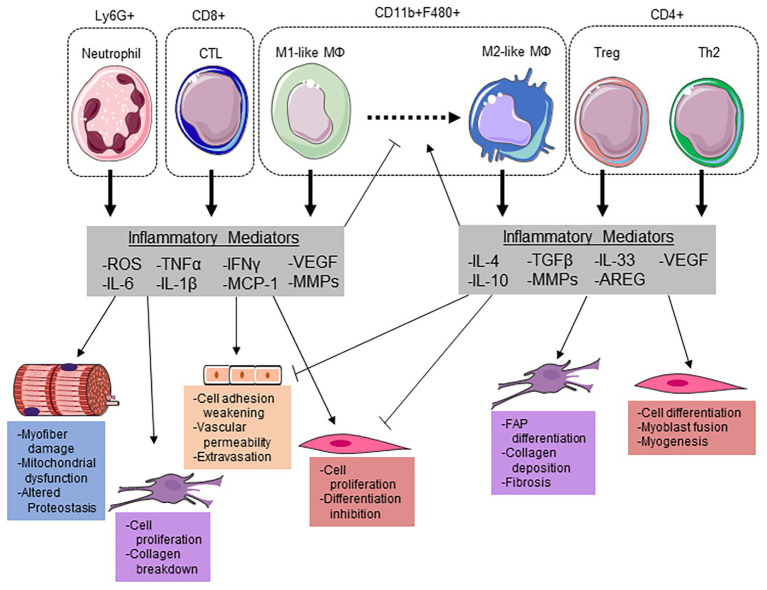
Immune cells modulate the myofiber microenvironment through releasing key inflammatory mediators. Neutrophils potentiate cellular damage through the release of reactive oxygen species (ROS), which can induce myofiber damage and increase vascular permeability for extravasation. Additionally, neutrophils release pro-inflammatory cytokines interleukin (IL)-6, IL-1β, and tumor necrosis factor α (TNF-α) to stimulate satellite cell proliferation and suppress differentiation. IL-6 and ROS have been demonstrated to induce mitochondrial dysfunction/damage and altered proteostasis within the myofiber. Along with cytotoxic T-lymphocytes (CTLs), neutrophils release monocyte chemoattractant protein (MCP) 1 to recruit inflammatory (M1-like) macrophages (MΦ). Both infiltrating monocytes and resident MΦs can polarize to an M1-like phenotype to aid in phagocytizing damaged myofibrillar proteins and the breakdown of collagen in the ECM through matrix metalloproteinases (MMPs). Along with an increase in Interleukin-4 (IL-4), regulatory T-cells (Tregs) can release IL-33 to stimulate amphiregulin (AREG) to promote MΦ polarization to a M2-like phenotype. These MΦs then release transforming growth factor (TGF) β and IL-10 to promote fibro-adipogenic progenitor (FAP) cell differentiation, ECM remodeling, and suppress the pro-inflammatory milieu. The reduction in pro-inflammatory cytokines allows for satellite cell differentiation and myogenesis. This figure was made with Servier Medical Art (http://www.servier.com/Powerpoint-image-bank).

The myofiber response to cancer and the cachectic environment is considered to be central to understanding muscle wasting regulation. However, there is an emerging consideration for tumor derived factors and inflammatory mediators to go beyond targeting the myofiber directly (Biswas and Acharyya, 2020). The cachectic environment has the potential to also indirectly disrupt myofiber homeostasis through the altered regulation of other cell types in the muscle microenvironment (Talbert and Guttridge, 2016), highlighting a need to delineate the capacity for cancer and chemotherapy to disrupt myofiber interactions with other cell types in muscle (Figure 2). Furthermore, each cell type has a critical function within the skeletal muscle microenvironment, involving ECM remodeling and angiogenesis, which are established components of muscle plasticity in response to regeneration from injury, growth, aging, overload-induced hypertrophy, and exercise (Dennis et al., 2009; Morgan and Partridge, 2020). Since skeletal muscle fiber nuclei are post-mitotic, the role of the satellite cell in aging and overload hypertrophy has been actively investigated for over 30 years. This line of inquiry includes the role of myonuclear apoptosis in sarcopenia and myonuclear accretion in hypertrophy myofibers (McCarthy et al., 2011; Fry et al., 2014). As skeletal muscle remodeling is a critical property of muscle and is thought to be a continuous process over an individual’s life span, there is growing interest in how aging, disuse, cancer, and chemotherapy treatments can disrupt remodeling processes in skeletal muscle, which could serve to exacerbate the development and progression of cachexia.

The ECM surrounding each muscle fiber can contain an assortment of fibroblasts, endothelial cells, immune cells, and satellite cells which are located beneath the basal lamina (Forcina et al., 2019). These cells have individual and coordinated roles in the response to local inflammation that accompanies muscle remodeling to damage, increased use/activity, and overload (Morgan and Partridge, 2020). Furthermore, changes within the ECM serve to initiate intracellular myofiber signaling through receptors on the sarcolemma in response to damage and mechanical stretch (Carson and Wei, 2000). The ECM is a rich source of growth factors and cytokines that can be released to regulate myofiber proteostasis and metabolic function (Forcina et al., 2019). Muscle fibers interact with the fibrous elements of the matrix through integrin receptors to sense mechanical signaling, and shifts in the expression of fibrous matrix proteins such as collagens have potential to alter intracellular signaling in muscle fibers (Carson and Wei, 2000). Thus, the cancer systemic environment has the potential to dysregulate myofiber gene expression and metabolic signaling through alterations to the ECM. Muscle fibrosis and dysregulation of the ECM has been reported in cachectic skeletal muscle from pancreatic cancer patients (Judge et al., 2018; Nosacka et al., 2020), and fibrosis can negatively impact myofiber growth and healing from damage (Bedair et al., 2007; Li et al., 2016a). Muscle inflammatory processes are established modulators of the ECM for healing and wound repair and also critical for muscle remodeling to exercise and overload. In fact, disruptions to IL-6 expression during overload-induced hypertrophy and during the recovery from disuse-induced muscle atrophy in mice can alter the hypertrophy response, collagen composition within the ECM, and muscle fibrosis in mice (White et al., 2009; Washington et al., 2011). Furthermore, during the initiation of compensatory overload-induced hypertrophy in mice an inflammatory and ECM gene expression is strongly induced (Carson et al., 2002). These observations demonstrate the complexity of the ECM response and local inflammation, since they drive hypertrophy with overload and healing from damage, but are also implicated in cancer-induced muscle wasting (Forcina et al., 2019). To this end, local inflammatory responses are subjected to precise temporal regulation and if this response is altered muscle remodeling can be either attenuated or blocked (Howard et al., 2020), and further research is warranted to determine if cancer can disrupt the temporal aspects of local inflammation needed for successful remodeling. For example, inflammatory signaling can impact several cell types located in the muscle microenvironment leading to altered myofiber protein synthesis (Gao et al., 2017) and mitochondrial quality control (Gomez-Cabrera et al., 2016), which are known drivers of muscle wasting with cancer. In this section, three important cell types are highlighted that interact with the myofiber within the muscle microenvironment and their potential role in cancer and chemotherapy-induced muscle wasting is emphasized.

### Satellite Cells

The role of the satellite cell in skeletal muscle physiological and biological responses to a variety of conditions, stimuli, and disease states has been extensively investigated since being first discovered by Mauro in 1961 (Mauro, 1961; Penna et al., 2010; He et al., 2013). The satellite cell is located between the myofiber sarcolemma and basal lamina and is not easily distinguished from a myonuclei with light microscopy. These cells remain in a quiescent state, but can be activated to proliferate and differentiate by a variety of growth factors and mitogenic stimuli (Bischoff, 1986a,b). Satellite cell differentiation can involve either fusion with existing myofibers to allow for myonuclear accretion or the formation of *de novo* myotubes. Therefore, the satellite cell has been extensively examined as a myogenic stem cell in skeletal muscle critical for muscle repair and growth processes. Both the regulation and disruption of satellite cell number, activation, proliferation potential, and differentiation have been widely investigated for 60 years (Forcina et al., 2019). Interestingly, one of the earliest described inducers of satellite cell proliferation was FGF, and this activation was also linked to damage disruption of the muscle ECM (Bischoff, 1986a,b). This line of inquiry coincides with investigation to this day involving satellite cell control by their microenvironment and the cells that are at the source of these mitogenic factors. There has been increasing interest in understanding the cancer environment’s effect on satellite cell activity, which could directly impact cachectic muscle’s diminished capacity for muscle regeneration (Schwarzkopf et al., 2006; Penna et al., 2010; He et al., 2013; Talbert and Guttridge, 2016). Deficits in the capacity for myogenesis, which is needed for successful muscle regeneration, have been reported in skeletal muscle from preclinical cachexia models and cancer patients, and involve an inability of satellite cells to effectively differentiate to complete the regenerative process (Penna et al., 2010; He et al., 2013). Due to the importance of the successful myogenic response in muscle regeneration, signaling pathways disrupting satellite cell activity have been actively investigated as potential therapeutic targets for cancer-induced muscle wasting (Schwarzkopf et al., 2006; He et al., 2013; Cerquone Perpetuini et al., 2018; Costamagna et al., 2020; Schmidt et al., 2020). To this end, skeletal muscle satellite cell differentiation has been reported to be rescued in colon-26 induced cachexia in mice through the inhibition or ERK signaling (Penna et al., 2010), and more recently IL-4 administration (Costamagna et al., 2020). Pax7 is a positive regulator of satellite cell proliferation, which has been reported to be disrupted in cachectic muscle from mice and cancer patients (He et al., 2013); dysregulation of Pax7 in cachectic muscle was driven by aberrant NF-κΒ signaling induced by circulating tumor derived factors. Wnt signaling is an established regulator of muscle satellite cell activity through the expression of myogenic regulatory factors and increased Wnt7a in colon-26 induced cachexia in mice can increase muscle regeneration by the improvement of muscle satellite regulation of differentiation and the induction of anabolic signaling through Akt/mTORC1 (Schmidt et al., 2020). Interestingly, mathematical modeling of colon-26 induced cachexia in mice recently predicted that blocking the myostatin/activin A pathway could attenuate cancer-induced muscle mass loss through the restoration of functional satellite cell differentiation (Farhang-Sardroodi and Wilkie, 2020). Depending on the extent of the damage, successful muscle regeneration including the initiation and resolution of local inflammatory processes involves infiltrating monocytes, resident MΦs, neutrophils, and T-cells (Morgan and Partridge, 2020). Local inflammatory responses are subjected to precise temporal regulation and if this response is altered muscle remodeling can be either attenuated or blocked (Howard et al., 2020), and further research is warranted to determine if cancer can disrupt the temporal aspects of local inflammation that contribute to disrupted satellite cell regulation of differentiation in cachectic muscle.

The satellite cell has been hypothesized to have critical roles in post-natal muscle growth, overload-induced hypertrophy, capacity of muscle regeneration after damage, and the development of sarcopenia. The development of modern molecular biology tools has shed light on the role of satellite depletion on these processes (McCarthy et al., 2011; Fry et al., 2014), but interesting gain of function experiments remain elusive. While the role of the satellite cell with some types of overload-induced growth and sarcopenia remains equivocal (Murach et al., 2018), the satellite cell has an established role in muscle regeneration from injury. Interestingly, there is emerging evidence that satellite cells have a regulatory role in ECM during overload-induced hypertrophy (Murach et al., 2018). Additionally, MΦs have been demonstrated to interact with satellite cells to regulate myogenesis (Dort et al., 2019). Pro-inflammatory monocytes and MΦs release cytokines, IL-6 and TNF-α, to stimulate satellite cell proliferation and inhibit differentiation until switching to an M2-like phenotype to promote differentiation and fusion (Arnold et al., 2007). T-cells have been demonstrated to maintain satellite cells myogenic potency *in vitro* (Fu et al., 2015), while T-cell deficient mice had reduced satellite cell proliferation contributing to blunted repair (Castiglioni et al., 2015; Deyhle and Hyldahl, 2018). There is significant interest in muscle microenvironment changes that occur with aging that could alter satellite cell regulation and serve to impede muscle regeneration from injury (Lee et al., 2019). Satellite cell number and activity are thought to be impacted by aging, and other regulators of satellite cell function such as the immune response are also aging targets (Forcina et al., 2019). Therefore, the impact of age on satellite cell activity could be a factor in the development and progression of cachexia due to the advanced age of many patients having cancers that are associated with high cachexia incidence. These facts could directly impact our current mechanistic understanding of cancer cachexia, as preclinical modeling that account for the effects of sarcopenia and aging on the skeletal muscle microenvironment are needed to answer these critical questions.

### Fibroblasts

Fibroblasts have a central role in the formation and remodeling of the skeletal muscle ECM which provides stability for the myofiber, blood vessels, and nerves (Chapman et al., 2016; Mendias, 2017). Fibroblasts aid in the synthesis and deposition of collagen, which predominate skeletal muscle connective tissues. Matrix metalloproteinases (MMPs) are largely responsible to the breakdown and deposition of collagen in the ECM. MMPs are released by immune cells, myofibers, satellite cells, epithelial cells, and fibroblasts themselves and are transcriptionally regulated by classic cytokine signaling cascades (Thomas et al., 1998; Liu et al., 2006; Phatharajaree et al., 2007; Yndestad et al., 2007; Iyer et al., 2016). These cells release MMPs to degrade several components of the cytoskeleton and stimulate *de novo* collagen deposition by the fibroblast (Sundararaj et al., 2009). Additionally, fibroblasts and satellite cells coordinate during skeletal muscle regeneration to limit fibrosis and promote healthy hypertrophic repair (Murphy et al., 2011).

The role of several immune cell types and inflammatory mediators in skeletal muscle fibroblast proliferation and collagen deposition has been extensively studied due to their integral functions in tissue repair (Van Linthout et al., 2014). In addition to providing structural support, fibroblasts are sensitive to mechanical stretch and maintain the ECM by aiding in the breakdown and synthesis/deposition of skeletal muscle collagens (Tomasek et al., 2002). Additionally, this mechanical sensitivity allows fibroblasts to release their own cytokines/chemokines for early neutrophil recruitment to aid in the breakdown of the damaged ECM through the action of MMPs, collagenases, and elastases (Baici et al., 1982; Kim and Luster, 2015); however, this mechanistic link in skeletal muscle requires additional work. On the other hand, innate immune cells can induce re-expression of fetal genes, activation of MMPs, fibro-adipogenic progenitor (FAP) differentiation, proliferation of fibroblasts, and myofibroblast formation (Tomasek et al., 2002; Gentek and Hoeffel, 2017; Tidball, 2017). Pro-inflammatory cytokines TNF-α, IL-6, and IL-1β are produced and secreted by Th cells and M1-like MΦs to stimulate fibroblast proliferation, while the anti-inflammatory TGFβ and IL-10 are predominately produced by Tregs and M2-like MΦs to stimulate fibroblast collagen synthesis and deposition (Van Linthout et al., 2014; Jablonski et al., 2015). M2-like MΦ release of arginase 1 (Arg1) also can stimulate fibroblast proliferation through the production of polyamines from arginase metabolism (Tidball, 2017). While the proper coordination of fibroblast proliferation and collagen formation is necessary for healthy skeletal muscle repair and remodeling, both reduced or exacerbated fibroblast activity can result in fibrosis and reduced skeletal muscle plasticity and overall functional capacity (Chapman et al., 2016). Fibrosis during the progression of cancer cachexia has received significant attention due to its role in reduced skeletal muscle plasticity and the loss of strength disproportionate to mass (Judge et al., 2018; Ramage and Skipworth, 2018); however, evidence of impaired regeneration and increased fibrosis in cachectic skeletal muscle is limited to cancer type and severity of the disease (Nosacka et al., 2020).

### Endothelial Cells

Endothelial cells are cells that are associated with blood vessels, which vascularize skeletal muscle. Intuitively, skeletal muscle vascularization plays central roles in nutrient transportation (e.g., glucose delivery and CO_2_ removal) and global/systemic communication (e.g., hormones and cytokines/myokines). When necessary, the myofiber, resident fibroblasts, and/or resident immune cells secrete cytokines and pro-inflammatory mediators to activate nearby endothelial cells initiating circulating immune cell extravasation into the damaged tissue (Vestweber, 2015). As previously discussed, the influx of neutrophils, activated monocytes, and pro-inflammatory MΦs are central for skeletal muscle repair and remodeling. A key function of neutrophils in promoting cell death is the generation of ROS through NADPH oxygenase-dependent mechanisms (Kolaczkowska and Kubes, 2013). While ROS can be destructive to skeletal muscle in several ways, ROS is an important regulator of skeletal muscle vascular permeability (Korthuis et al., 1985). VEGF has received significant attention for its role in stimulating angiogenesis and promotion of vascular permeability is vital for skeletal muscle repair and remodeling (Arsic et al., 2004). Endothelial cells rely heavily on self VEGF production and autocrine signaling for vascular homeostasis and this autocrine signaling cascade differs from exogenous or paracrine VEGF signaling (Lee et al., 2007). Interestingly, VEGF has been implicated as a regulator of DC differentiation and MΦ polarization (Li et al., 2016b). While monocytes and MCP-1 stimulate the release of VEGF by endothelial cells to increase myofibrillar vascularization during the later stages of repair (Hong et al., 2005), tumor associated macrophages (TAMs) promote the release of VEGF to promote tumor vascularization (Wynn et al., 2013). However, IL-4 inhibits basic Fibroblast Growth Factor (bFGF) induced angiogenesis (Volpert et al., 1998). Therefore, it is vital to understand off-target effects of modulating immune cells and their inflammatory mediators on the endothelium within the skeletal muscle microenvironment.

Endothelial cells have sustained significant attention in tumor cell biology given the importance of tumor vascularization in tumor cell growth and metastasis (Dudley, 2012; Li et al., 2016b). Naturally, treatments targeting endothelial cells and mitigating vascularization has emerged as a promising therapy; however, given the importance of vascular homeostasis in maintaining healthy tissue, the off-target implications of these treatments invoke caution (Morabito et al., 2006). While the effects of cachexia and chemotherapy on the skeletal muscle endothelium or the relationship between immune cells and the skeletal muscle endothelium has not been directly studied, we can glean information from studies examining aging, non-cancer-induced cachexia, and established inflammatory signaling and nutrient availability mechanisms. For instance, COPD patients suffering from cachexia have reduced skeletal muscle capillary density partly caused by deconditioning (Jobin et al., 1998); given that cancer patients experience fatigue and reduced activity, we can expect that cachectic skeletal muscle with cancer has decreased vascularization. Reduced vascularization would diminish skeletal muscle’s regenerative capacity resulting in increased fibrosis, reduced strength, and fatigue (Arsic et al., 2004; Allen et al., 2008; Inaba et al., 2018; Judge et al., 2018; Vanderveen et al., 2018). Additionally, aging processes contributing to frailty has been associated with endothelial dysfunction (Argiles et al., 2016). Inhibition of angiogenesis is a critical target for some chemotherapeutics aimed at preventing vascularization of solid tumors through suppressing VEGF (Zhang et al., 2002; Shih and Lindley, 2006; Ooyama et al., 2008; Bozzetti, 2020). The impact on non-cancerous tissues continues to be unearthed; however, endothelial dysfunction has been demonstrated during chemotherapy driven cardiotoxicity (Brown et al., 2012). Additionally, anti-angiogenic therapies should aim to achieve vascular normalization in order to maintain the delivery and anti-tumor efficacy of chemotherapeutics as opposed to eliminating tumor vascularity completely (Viallard and Larrivee, 2017). Chemotherapy’s effect on skeletal muscle’s endothelium has not been directly investigated. Given the neutrophil’s role in ROS generation, the neutropenia associated with chemotherapy may inhibit the skeletal muscle’s ability to increase vascular permeability and immune cell extravasation. Further work is needed to understand the impact of cancer and its treatments on skeletal muscle’s vascular endothelium.

### Other Cells Within the Skeletal Muscle Microenvironment: The Motor Unit

The nervous and musculoskeletal systems are inextricably linked through the motor unit and denervation, or the loss of the neuromuscular junction (NMJ), results in loss of function and rapid muscle atrophy (Bonaldo and Sandri, 2013). Denervation results in the affected myofibers losing their contractile capacity, which is then followed by a marked reduction in myofibrillar cross sectional area. If the NMJ is not restored, the affected myofibers will become unrecognizable, as they lose their sarcomeric structure and become fibrotic (Carlson, 2014). Similar to other atrophic conditions, MΦs have been identified as contributors to denervation-induced fibrosis (Mochizuki et al., 2005). Interestingly, denervation with cancer cachexia has been suggested in C26 mice (Daou et al., 2020), and neurotoxicity was observed with chemotherapeutic Folfiri (Barreto et al., 2016a); however, gastrointestinal cancer patients with cachexia had no significant loss of NMJs compared to weight stable or health controls (Boehm et al., 2020). Chemotherapeutic DOX disrupted the NMJ in sedentary rats while exercise was able to improve neuromuscular gene and protein expression (Huertas et al., 2020).

## Conclusions and Future Directions

The cellular environment surrounding the myofiber, or the ECM, is a mixture of mononucleated satellite cells, fibroblasts, endothelial cells, and immune cells. Together these cells cooperate and aid in locomotion (e.g., tendon formation/strength), systemic and local metabolic homeostasis, structural support, repair/remodeling, and systemic communication (e.g., myokines). Coordination of this complex network relies on each cell functioning to communicate within endocrine, paracrine, and autocrine mechanisms given a multitude of stimuli (Meng et al., 2015; Bersini et al., 2018). The complex nature of the skeletal muscle microenvironment invites difficulty in identifying key regulators of muscle mass and function loss with cancer and chemotherapy; however, targeting inflammation and immune cells prior to‐ or during cachexia’s progression could promote widespread benefits given their regulatory roles in several skeletal muscle processes. While several inflammatory mediators (IL-6, TNF-α, TWEAK, TRAF6, INF-γ, and LIF) have been implicated as drivers of cancer-associated wasting, very little is known about their origin, whether skeletal muscle, immune cell, or tumor-derived (Flint et al., 2016; Jackman et al., 2017). In this review, we highlighted that resident and circulating immune cells can modulate several aspects of the skeletal muscle microenvironment through a multitude of mechanisms. To this end, omitting investigation of multiple cell types within the muscle’s microenvironment during cachexia has the potential to hamper therapeutic developments/advancements leading to the current treatments, which have been unable to improve muscle function. Additional mechanistic inquiry is needed to understand how immune cells contribute to cancer and chemotherapy associated muscle mass and function loss.

For decades now, endurance and resistance exercise have been proposed as promising therapies as they broadly impact chronic inflammation, metabolic homeostasis, and muscle protein turnover (Al-Majid and McCarthy, 2001; Montalvo et al., 2018). There are barriers to exercise as a therapeutic for cancer cachexia as it is unlikely that a level of exercise to induce muscle mass can be performed by patients with debilitating conditions like cancer. However, muscle contractions induced by neuromuscular electrical stimulation was recently shown to improve muscle mass in breast cancer patients receiving chemotherapy (Guigni et al., 2019; Toth et al., 2020). Both resistance (Bredahl et al., 2020) and endurance (Smuder, 2019; Huertas et al., 2020; Montalvo et al., 2020) exercise have shown promise in mitigating chemotherapy-induced skeletal muscle toxicities in preclinical rats. Additionally, resistance (Hardee et al., 2016, 2018) and endurance (Puppa et al., 2012; Vanderveen et al., 2020) exercise can improve muscle mass and function in cachectic mice. Exercise also showed promise in preventing the loss of muscle mass and strength in C26 mice given 5FU + Oxaliplatin (Ballaro et al., 2019). Exercise oncology continues to be an active and intriguing field of study aimed at improving cancer patient’s survival and quality of life (Schmitz et al., 2019). While traditional nutritional support has been unsuccessful, several nutraceuticals and nutritional anti-inflammatories have promise in mitigating cancer-induced wasting (Narsale et al., 2016). Moreover, there is growing interest in identifying plant-derived compounds to treat cachexia given that they can modulate multiple pathways, are inexpensive, and have low toxicity as prolonged treatments (Saklani and Kutty, 2008). Plant-derived compounds have been shown to regulate inflammation through the modulation of MΦ polarization with cancer (Jia et al., 2014; Hu et al., 2017); however, the therapeutic efficacy of modulating MΦ phenotype and function during cancer-associated wasting has not been investigated.

## Author Contributions

JC planned, wrote, and edited manuscript. BV planned, wrote, and edited manuscript, created figures. EM edited and wrote manuscript. All authors contributed to the article and approved the submitted version.

### Conflict of Interest

BV and EM were employed by company AcePre, LLC.The remaining author declares that the research was conducted in the absence of any commercial or financial relationships that could be construed as a potential conflict of interest.
